# The Effect of Transcutaneous Electrical Acupoint Stimulation on Inflammatory Response in Patients Undergoing Limb Ischemia-Reperfusion

**DOI:** 10.1155/2017/8369737

**Published:** 2017-07-19

**Authors:** Yunchang Mo, Sijia Chen, Lili Yang, Ledan Huang, Dan Jin, Zhi Yu, Leilei Wang, Liangrong Wang, Shan Luo, Junlu Wang

**Affiliations:** Department of Anesthesia, The First Affiliated Hospital of Wenzhou Medical University, Wenzhou, Zhejiang 325000, China

## Abstract

Reperfusion after tourniquet use can induce inflammation and cause remote organ injury. We evaluated the therapeutic effect of transcutaneous electrical acupoint stimulation (TEAS) on inflammatory mediators and lung function in patients receiving lower limb tourniquets. Forty patients undergoing unilateral lower extremity surgery with tourniquet were randomly assigned to two groups: the TEAS group and ischemia-reperfusion (I/R) group. The C-C motif chemokine ligand 2 (CCL2), C-X-C motif chemokine ligand 8 (CXCL8), interleukin-1 (IL-1), interleukin-6 (IL-6), interleukin-10 (IL-10), tumor necrosis factor-*α* (TNF-*α*), and arterial blood gas analysis were measured preoperatively and 6 h after tourniquet removal. The levels of CXCL8, IL-1, IL-6, TNF-*α*, and CCL2 were significantly increased compared to baseline values in both groups, but the increase was significantly smaller in the TEAS group. In the TEAS group, the partial pressure of oxygen and arterial-alveolar oxygen tension ratio were significantly decreased, and the alveolar-arterial oxygen tension difference and respiratory index were significantly increased, compared to those in the I/R group at 6 h after reperfusion. In conclusion, TEAS diminished the upregulation of proinflammatory factors in response to lower limb ischemia-reperfusion and improved pulmonary gas exchange.

## 1. Introduction

Lower limb ischemia can be caused by a variety of clinical conditions, including critical limb ischemia, abdominal aortic aneurysm, and traumatic arterial injury [1]. Therapies that can restore perfusion to the ischemic limb are performed to reduce the injury caused by ischemia. However, reperfusion of the ischemic limb can induce inflammation and cause remote organ injury [2]. The lungs are some of the most vulnerable organs to insult subsequent to ischemia-reperfusion [3–5], which has been proved by models and clinical tests [6–8].

Tourniquets are typically used in orthopedic surgery to reduce intraoperative blood loss and decrease the allogeneic blood transfusion rate, thus providing clear vision for the surgery. This condition may be much more important in orthopedic procedures in which tourniquet-related ischemia-reperfusion damage develops and the lung gets injured [9]. Our previous research showed that tourniquet-induced lung injury was correlated with impaired gas exchange and that inflammation factors such as interleukin-1 (IL-1), interleukin-6 (IL-6), and tumor necrosis factor-*α* (TNF-*α*) are important mediators of this effect [10]. This suggests that reducing the inflammatory response should have a positive effect on postoperative recovery. The inflammatory factors are released as a result of the impairment of endothelial function and neutrophil infiltration during deflation of the tourniquet [8].

Acupuncture has been proven to exert anti-inflammatory effects in some diseases, such as asthma [11], rhinitis [12], and inflammatory bowel disease [13]. Acupuncture can treat these diseases by reducing the release of inflammatory substances. However, the anti-inflammatory action of acupuncture has not yet been confirmed by large randomized trials. For instance, Tian et al. demonstrated that acupuncture stimulation at ST-36 decreased the production of TNF-*α* in rats with ulcerative colitis [14]. Acupuncture preconditioning also has certain protective effects on different organs; in particular, its anti-inflammatory effects can protect against lung injury [15]. Song et al. also reported that acupuncture stimulation at ST-36 could reduce severe thermal injury-induced remote acute lung injury in rats [16].

To date, the question of whether transcutaneous electrical acupoint stimulation (TEAS) can protect lung tissue against the adverse effects of ischemia-reperfusion of the lower limbs remains unstudied. In this study, we administered TEAS to lower extremity surgery patients with tourniquets, observed the resulting changes in the inflammatory response, and assessed the pulmonary function by blood gas analysis.

## 2. Patients and Methods

### 2.1. Study Design and Patient Groups

**Figure 1:**
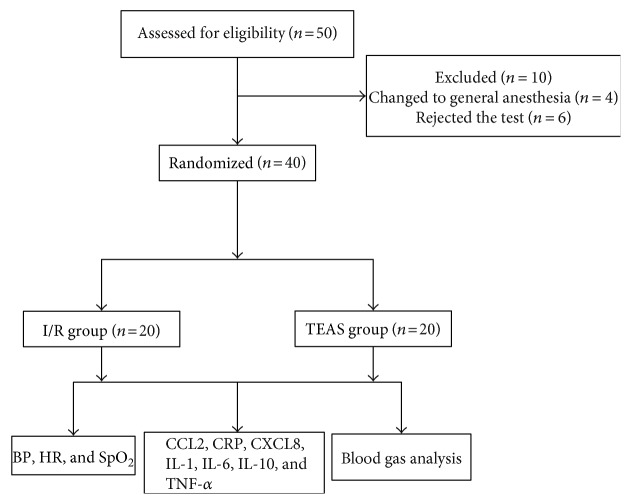
Flowchart of study design. I/R: ischemia-reperfusion, TEAS: transcutaneous electrical acupoint stimulation, BP: noninvasive blood pressure, HR: heart rate, SpO_2_: capillary oxygen saturation, CCL2: C-C motif chemokine ligand 2, CRP: C-reactive protein, CXCL8: C-X-C motif chemokine ligand 8, IL-1: interleukin-1, IL-6: interleukin-6, IL-10: interleukin-10, and TNF-*α*: tumor necrosis factor-*α*.

This randomized, double-blind, placebo-controlled clinical study (clinical trial registration number: ChiCTR-INR-17010499) was approved by the Ethics Committee of the First Affiliated Hospital of Wenzhou Medical University. All participants were informed of the study purpose and design and provided written consent prior to study enrolment. Initially, 50 lower limb surgery patients were selected. They were aged 20 to 65 years old, were of the American Society of Anesthesiologists (ASA) classes I-II, and tourniqueted for 60–90 minutes. The exclusion criteria were as follows: cardiopulmonary dysfunction; taken oxidant and antioxidant drugs within 1 week before surgery; and refused spinal and epidural anesthesia. Ten patients were excluded because of these criteria (Figure 1). Using a random data table, the remaining 40 patients were divided equally into the ischemia-reperfusion group (I/R group) and the transcutaneous electrical acupoint stimulation group (TEAS group).

### 2.2. TEAS Protocol

The acupoints ST36 and SP6 were chosen in the TEAS group. The TEAS was started at 30 minutes preoperatively and continued until the end of surgery. The parameters for TEAS were as follows: dilatational wave, 2/15 Hz, and strength, maximum tolerable intensity. The I/R group received acupuncture at the same acupoints but did not receive stimulation.

### 2.3. Anesthesia

The patients underwent routine preoperative preparation, including fasting for at least 8 h, establishing venous access, and regular monitoring of the electrocardiogram (ECG), noninvasive blood pressure (BP), heart rate (HR), and peripheral capillary oxygen saturation (SpO_2_). Patients were anesthetized by combined spinal and epidural block performed in L3-4 with 3 mL of 0.5% bupivacaine. The block was maintained at approximately the level of T8 for the duration of the surgery. To maintain circulatory stability, Ringer's lactate solution and hydroxyethyl starch injection were infused during the operation. Postoperative infusion of Ringer's lactate solution was performed to meet physiological requirements.

### 2.4. Data Collection

The mean arterial pressure (MAP) and heart rate (HR) were recorded before anesthesia (T0), 5 min after anesthesia (T1), 1 min before removing the tourniquet (T2), and at 1 min (T3), 5 min (T4), and 6 h (T5) after removing the tourniquet. In addition, a 2 mL radial artery blood sample was collected at T0 and T5: 1 mL for the gas analysis and the other 1 mL for the immunoassay (ELISA).

### 2.5. Blood Gas Analysis

The radial artery blood gas analysis measured the arterial partial pressure of oxygen (PaO_2_) and carbon dioxide (PaCO_2_), as well as hemoglobin (Hb) content. The derived variables included the arterial-alveolar oxygen tension ratio (a/A ratio), alveolar-arterial oxygen tension difference (A-aO_2_), and respiratory index (RI).

### 2.6. Enzyme-Linked Immunosorbent Assay (ELISA)

Arterial blood (1 mL) was collected at T0 and T5. Plasma was separated by centrifugation at 2500 rpm for 10 min and stored at −80°C for further analysis. The serum concentration of C-reactive protein (CRP), TNF-*α* (tumor necrosis factor-*α*), CCL2 (C-C motif chemokine ligand 2), CXCL8 (C-X-C motif chemokine ligand 8), IL-1 (interleukin-1), IL-6 (interleukin-6), and IL-10 (interleukin-10) were measured using a human ELISA kit (Jiancheng Co., Nanjing, China), according to the manufacturer's instructions.

### 2.7. Statistical Analysis

All statistical analyses were performed using SPSS, version 13.0 (SPSS Inc., IL, USA). The discrete variables were analyzed with the chi-square test, repeated measurements were compared using repeated measures' analysis of variance (ANOVA), and continuous variables were compared using the Student's *t*-test. Data were expressed as mean ± standard deviation. For all tests, *p* < 0.05 was considered statistically significant.

## 3. Results

### 3.1. Clinical Characteristics of Patients

All forty patients completed the study. There were no significant differences in clinical characteristics between the two groups, including gender, age, height, weight, operation time, and tourniquet duration (*p* > 0.05; Table 1).

### 3.2. Hemodynamic Parameters

As shown in Table 2, there were no significant differences in MAP or HR between the two groups (*p* > 0.05).

### 3.3. Blood Gas Analysis

In the I/R group, the PaO_2_ and a/A ratio were significantly decreased, and the A-aO_2_ and respiratory index were significantly increased at T5, compared to T0 (*p* < 0.05). At T5, the PaO_2_ and a/A ratio were significantly higher, and the A-aO_2_ and respiratory index were significantly lower in the TEAS group, compared with those in the I/R group (*p* < 0.05; Table 3).

### 3.4. Inflammatory Cytokine and CRP Levels

As shown in Table 4, in the I/R group, the IL-1, IL-6, and CCL2 levels at T5 were significantly higher, compared with those at T0 (*p* < 0.05). Meanwhile, the levels of IL-1, IL-6, CXCL8, CCL2, and TNF-*α* were significantly lower in the TEAS group at T5, compared to those in the I/R group at T5 (*p* < 0.05). There was no significant difference in CRP and IL-10 between time points or groups (*p* > 0.05).

## 4. Discussion

In the present study, we demonstrated that limb ischemia-reperfusion can lead to (1) a systemic inflammatory response, represented by an increase of proinflammatory factors like IL-6 and CCL2, and (2) impaired gas exchange in the lungs, demonstrated by decreased PaO_2_, A-aO_2_, and RI. Importantly, we found that TEAS could mitigate these effects.

Although tourniquets play an important role in orthopedic surgery, there are several potential local and systemic complications associated with tourniquet use, including tissue edema, pain, and injuries of remote sites like the heart, lungs, liver, kidney, brain, and other organs [17]. Lung injury can result from changes in capillary permeability and neutrophil infiltration, which lead in turn to impaired lung function [18]. Using a rat model of ischemia-reperfusion, Kalb et al. found a large amount of neutrophil aggregation in the lungs [19]. Another study using an intestinal ischemia-reperfusion model also found lung injury and a significantly lower a/A ratio [20].

### 4.1. Blood Gas Analysis

The a/A ratio, A-aO_2_, and respiratory index can be used to assess pulmonary gas exchange. We found that the a/A ratio, A-aO_2_, and respiratory index were significantly decreased at 6 h after tourniquet release, implying that gas exchange in the lungs was impaired, similar to our findings from a previous study [10]. In addition, we found that these indices were improved by TEAS.

### 4.2. Inflammatory Cytokine Levels

The mechanism of remote injury due to ischemia-reperfusion of the limbs, especially in the lungs, has not been fully elucidated. However, the inflammatory response has been implicated as an important mediator of lung injury after limb ischemia-reperfusion. Cell damage and stress lead to the activation of inflammatory cells such as macrophages and neutrophils, which can release a large number of inflammatory cytokines, including IL-1, IL-6, TNF-*α*, and CXCL8 [21]. The CRP was also monitored in all patients, and there was no statistical difference between the two groups before and after the operation, indicating that there was no difference in the inflammatory response between the patient groups.

#### 4.2.1. IL-1

IL-1 is an acute inflammatory factor that is involved in the process and regulation of ischemia-reperfusion injury. Shih et al. established a mouse model of limb ischemia-reperfusion injury and found that IL-1 mRNA expression in blood was significantly increased at 4 hours after reperfusion [22]. Our study also found that IL-1 levels were significantly increased at 6 hours after reperfusion in the I/R group and that TEAS could significantly reduce this.

#### 4.2.2. IL-6

Studies have shown that the peripheral blood concentration of IL-6 is proportional to the degree of ischemia-reperfusion injury; it was a predictor of the severity of tissue damage and was found to correlate with an increasing incidence of complications [23, 24]. In the present study, IL-6 levels were significantly elevated at 6 h after tourniquet removal in both groups, but the increase in the TEAS group was significantly less than that in the I/R group.

#### 4.2.3. CXCL8

CXCL8 is also named interleukin-8 (IL-8), which is a proinflammatory response factor that is produced by a variety of cells, such as monocytes, macrophages, and T cells. CXCL8 levels can be significantly increased after ischemia-reperfusion, likely due to cell damage and stress caused by neutrophils, monocytes, macrophages, and other inflammatory cells. A previous study also confirmed that serum CXCL8 levels were significantly increased at 2, 6, and 24 hours after reperfusion in patients with limb ischemia-reperfusion injury [25]. In this study, we also found that CXCL8 levels were elevated at 6 hours after reperfusion, and the TEAS group showed significantly lower CXCL8 levels at 6 hours after reperfusion than the I/R group.

#### 4.2.4. TNF-*α*

TNF-*α* is one of the earliest released and most sensitive inflammation factors [8]. It can induce the production of IL-6, further stimulating the inflammatory response. Anti-TNF antibodies have been shown to reduce pulmonary injury following lower limb ischemia-reperfusion [26, 27]. In the present study, TNF-*α* levels were significantly higher at T5 in the I/R group as compared to the TEAS group.

#### 4.2.5. CCL2

CCL2 is a basophil chemotactic and activating agent, that is, it stimulates basophil degranulation and histamine release [28, 29]. CCL2 is also named monocyte chemotactic protein 1 (MCP-1), which is one factor of the inflammatory responses, and controls the production and release of other inflammatory mediators. Furthermore, CCL2 plays a critical role in attracting a variety of inflammatory cells, especially the mononuclear cells, to gather at affected areas. Abbruzzese et al. showed that CCL2 levels were increased after reperfusion in a mouse model of limb ischemia-reperfusion [30]. As was the case with IL-6 and TNF-*α*, the levels of CCL2 were increased after reperfusion in both groups, but the increase in the TEAS group was significantly less than that in the I/R group.

#### 4.2.6. IL-10

IL-10 is an anti-inflammatory factor that inhibits the production of IL-6 and TNF-*α*. A moderate amount of IL-10 inhibits the secretion of inflammatory mediators and promotes the release of anti-inflammatory factors [31]. We did not observe any significant differences in IL-10 levels in the present study, suggesting that the effect of TEAS is restricted to proinflammatory agents.

### 4.3. Effects of Acupuncture and TEAS

Studies have shown that acupuncture functions as a stimulus signal that is transmitted through the sciatic nerve to produce anti-inflammatory effects in the central nervous system [32]. Studies also have shown that electric acupuncture at zusanli (ST36) and sanyinjiao (SP6) has anti-inflammatory and organ-protective effects [16, 33], which were correlated with vagus nerve excitement. Torres-Rosas et al. found that acupuncture at ST36 reduced the levels of IL-6 and TNF-*α* in sepsis mice, thus reducing postoperative infection and improving the survival rate [32]. Furthermore, animal experiments and clinical studies have demonstrated that acupuncture at ST36 can reduce the levels of abnormally increased proinflammatory factors during the inflammatory response [16, 34].

Acupuncture can also play an important anti-inflammatory role in different diseases [13]. In a rat model of neuropathic pain, Cha et al. found that electroacupuncture could reduce the expression of proinflammatory factors and reduce the amount of pain [35]. In other inflammation-related disease models, such as rheumatoid arthritis [36], ulcerative colitis, and achronic stress models of depression [37], acupuncture has also been found to reduce the levels of inflammatory factors and alleviate the disease symptoms [38].

At present, ST36 and SP6 are two of the most recognized acupuncture points with clear anti-inflammatory effects [32]. In the present study, we found that patients who received TEAS at these points had significantly lower levels of IL-6, TNF-*α*, and CCL2 at 6 h after reperfusion. In addition, we found that IL-10 levels were not different between time points or groups. Guo et al. found a similar result in a rat model of acute pancreatitis [39]. This suggests that TEAS may play a critical role in anti-inflammation through reducing the levels of proinflammatory cytokines in the plasma but may not have much influence on anti-inflammatory cytokines.

## 5. Conclusions

Although short-term tourniquet use is generally considered safe, we found that lower extremity surgery patients who received a tourniquet exhibited an increase in the levels of proinflammatory cytokines and impaired lung function. Although there were no complications in the present group, patients with more risk factors, such as the elderly, and those experiencing cardiopulmonary dysfunction, multiple injuries, emergency operation, or a delayed tourniquet duration, might be more susceptible to reperfusion injury. TEAS at ST36 and SP6 was found to mitigate these effects; it is a minimally invasive intervention that can be safely and effectively applied in clinical settings.

## Figures and Tables

**Table 1:** Clinical characteristics of the patients (*n* = 20 in each group). Data are expressed as mean ± standard deviation.

Group	Gender (M/F)	Age (y)	Height (cm)	Weight (kg)	Operation time (min)	Tourniquet duration (min)
I/R	13/7	42 ± 12.4	168 ± 5.9	61.6 ± 7.8	91.5 ± 14.7	79.5 ± 10.1
TEAS	12/8	43.9 ± 14.4	165.6 ± 7.2	63.3 ± 8.5	90.4 ± 20.0	80.7 ± 11.7

I/R: ischemia-reperfusion; TEAS: transcutaneous electrical acupoint stimulation.

**Table 2:** Mean arterial pressure and heart rate of the two groups (*n* = 20 in each group). Data are expressed as mean ± standard deviation.

	Groups	T0	T1	T2	T3	T4	T5
MAP (mmHg)	I/R	96 ± 7	91 ± 4	90 ± 6	89 ± 6	92 ± 8	91 ± 5
	TEAS	92 ± 5	91 ± 4	92 ± 4	91 ± 4	92 ± 6	92 ± 4
HR (bpm)	I/R	78 ± 14	80 ± 13	77 ± 11	83 ± 15	74 ± 9	76 ± 9
	TEAS	77 ± 10	77 ± 9	78 ± 12	81 ± 9	76 ± 11	73 ± 9

HR: heart rate; I/R: ischemia-reperfusion; MAP: mean arterial pressure; TEAS: transcutaneous electrical acupoint stimulation.

**Table 3:** Blood gas analysis (*n* = 20 in each group). Data are expressed as mean ± standard deviation.

	Group	T0	T5
PCO_2_	I/R	38.85 ± 3.06	39 ± 3.15
	TEAS	37.55 ± 2.93	38.8 ± 3.27
PaO_2_	I/R	92.7 ± 5.58	81.75 ± 5.03^∗^
	TEAS	93.65 ± 5.87	86.65 ± 7.23^∗^^,#^
A-aO_2_	I/R	8.47 ± 5. 39	19.23 ± 7.06^∗^
	TEAS	9.14 ± 6.57	14.13 ± 7.52^∗^^#^
a/A ratio	I/R	91.68 ± 5.21	81.12 ± 6.58^∗^
	TEAS	91.20 ± 6.29	85.78 ± 8.58^∗^^,#^
RI	I/R	9.43 ± 6.49	24.03 ± 9.91^∗^
	TEAS	10.14 ± 7.37	16.91 ± 9.79^∗^^,#^
Hb	I/R	10.88 ± 1.10	10.555 ± 0.90
	TEAS	10.95 ± 1.26	10.77 ± 1.25

^∗^ *p* < 0.05 versus T0. ^#^*p* < 0.05 versus the I/R group. a/A ratio: arterial-alveolar oxygen tension ratio; A-aO_2_: alveolar-arterial oxygen tension difference; Hb: hemoglobin; I/R: ischemia-reperfusion; PaCO_2_: arterial partial pressure of carbon dioxide; PaO_2_: arterial partial pressure of oxygen; RI: respiratory index; TEAS: transcutaneous electrical acupoint stimulation.

**Table 4:** Plasma CRP, CCL2, CXCL8, IL-1, IL-6, TNF-*α*, and IL-10 levels (*n* = 20 in each group). Data are expressed as mean ± standard deviation.

	Groups	T0	T5
CRP (pg/mL)	I/R	7.9 ± 1.2	8.4 ± 1.6
	TEAS	7.7 ± 2.0	8.0 ± 1.9
CCL2 (pg/mL)	I/R	108.8 ± 38.8	154.2 ± 42.9^∗^
	TEAS	107.1 ± 31.0	111.7 ± 50.0^#^
CXCL8 (pg/mL)	I/R	8.1 ± 3.0	24.2 ± 6.2^∗^
	TEAS	8.5 ± 3.4	16.3 ± 4.2^∗^^,#^
IL-1 (pg/mL)	I/R	18.6 ± 4.4	31.4 ± 9.3^∗^
	TEAS	17.7 ± 4.3	23.9 ± 7.5^∗^^,#^
IL-6 (pg/mL)	I/R	6.1 ± 2.0	61.6 ± 4.5^∗^
	TEAS	5.4 ± 1.0	30.4 ± 4.5^∗^^,#^
TNF-*α* (pg/mL)	I/R	16.7 ± 6.0	20.8 ± 8.9
	TEAS	17.2 ± 7.7	12.0 ± 4.7^∗^^,#^
IL-10 (pg/mL)	I/R	2.5 ± 1.0	2.8 ± 1.2
	TEAS	2.9 ± 1.2	3.6 ± 1.3

^∗^ *p* < 0.05 versus T0. ^#^*p* < 0.05 versus the I/R group. I/R: ischemia-reperfusion; CRP: C-reactive protein; CCL2: C-C motif chemokine ligand 2; CXCL8: C-X-C motif chemokine ligand 8; IL-1: interleukin-1; IL-6: interleukin-6; IL-10: interleukin-10; TEAS: transcutaneous electrical acupoint stimulation; TNF-*α*: tumor necrosis factor-*α*.
